# Supplementary figures and images for: Combining diaries and accelerometers to explain change in physical activity during a lifestyle intervention for adults with pre-diabetes: A PREVIEW sub-study
Source: PLoS One. 2024 Mar 21;19(3):e0300646. doi: 10.1371/journal.pone.0300646 (PMC10956823; doi:10.1371/journal.pone.0300646)

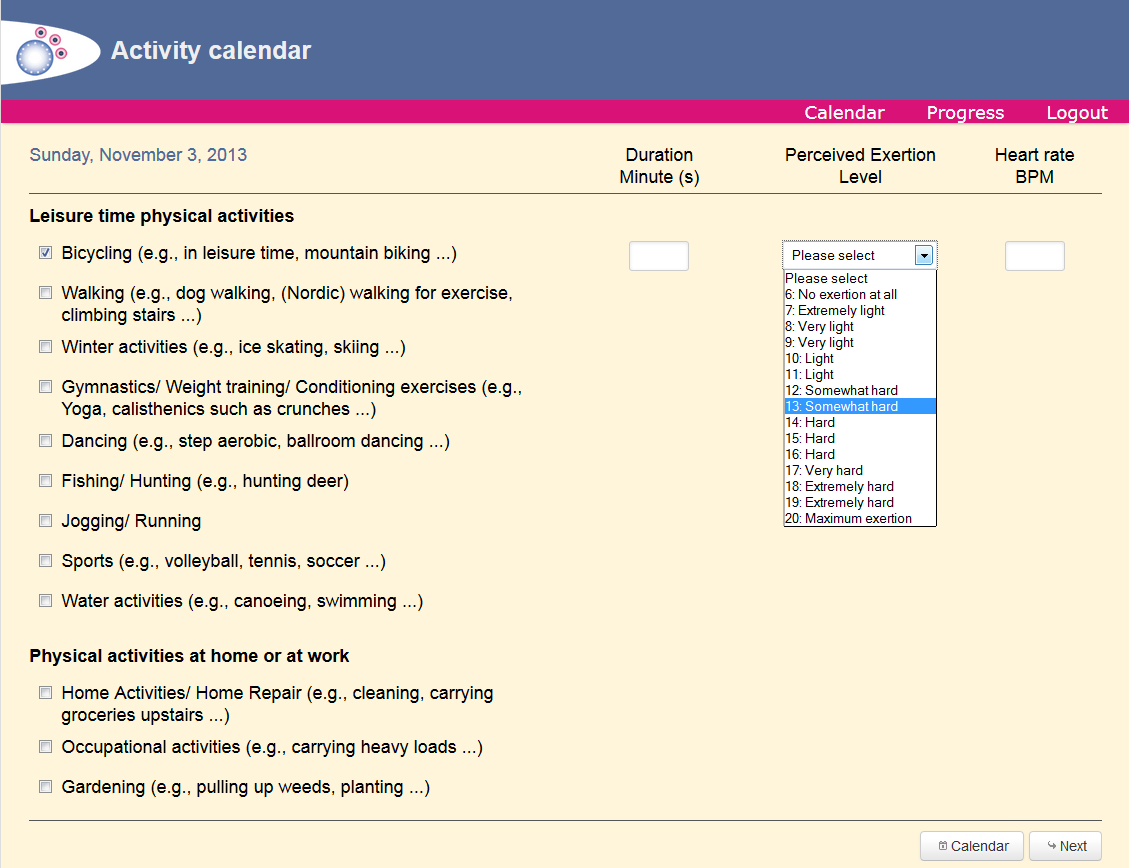

Supplement: S1 Fig — (TIF) [file pone.0300646.s001.tif]

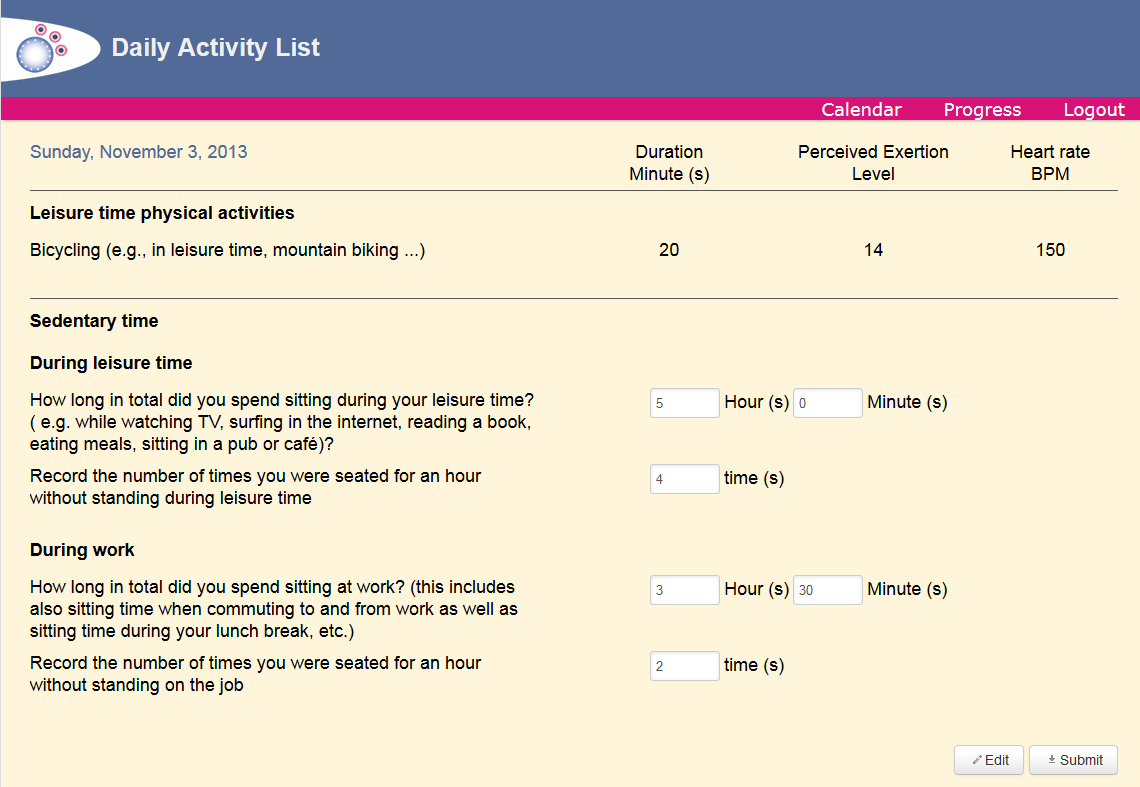

Supplement: S2 Fig — (TIF) [file pone.0300646.s002.tif]
