# Supplementary material for: Combining diaries and accelerometers to explain change in physical activity during a lifestyle intervention for adults with pre-diabetes: A PREVIEW sub-study
Source: PLoS One. 2024 Mar 21;19(3):e0300646. doi: 10.1371/journal.pone.0300646 (PMC10956823; doi:10.1371/journal.pone.0300646)
Supplement: S1 Table — 1 Linear Model ANOVA, 2 Pearson’s Chi-squared test, PA—physical activity, BMI–Body Mass Index, BL—Baseline, PAL–physical activity level; SED–sedentary time, LPA–light physical activity; MVPA–moderate-to-vigorous physical activity. (DOCX) [file pone.0300646.s003.docx]

**S1 Table. Comparison of included and excluded participants in the longitudinal PA diary sample.**

|  | Full PREVIEW Sample (excluding PA diary sample) (n = 1,991) | Longitudinal PA diary sample (N = 232) | p value |
| --- | --- | --- | --- |
| Age (at baseline) |  |  | < 0.001^1^ |
| - Mean (SD) | 51.76 (11.73) | 55.83 (9.54) |  |
| Sex |  |  | 0.144^2^ |
| - Female | 1,356 (68.1%) | 147 (63.4%) |  |
| - Male | 635 (31.9%) | 85 (36.6%) |  |
| BMI (at BL) |  |  | < 0.001^1^ |
| - n | 1,988 | 232 |  |
| - Mean (SD) | 35.65 (6.71) | 32.82 (4.53) |  |
| BMI (at 12 months) |  |  | < 0.001^1^ |
| - n | 1,035 | 232 |  |
| - Mean (SD) | 31.49 (5.88) | 29.28 (4.39) |  |
| Country |  |  | < 0.001^2^ |
| - Denmark | 337 (16.1%) | 42 (18.1%) |  |
| - Finland | 192 (9.2%) | 97 (41.8%) |  |
| - Netherlands | 181 (8.6%) | 24 (10.3%) |  |
| - UK | 256 (12.2%) | 8 (3.4%) |  |
| - Spain | 261 (12.5%) | 46 (19.8%) |  |
| - Bulgaria | 368 (17.6%) | 0 (0.0%) |  |
| - Australia | 194 (9.3%) | 0 (0.0%) |  |
| - New Zealand | 305 (14.6%) | 15 (6.5%) |  |
| PA intervention group |  |  | 0.483^2^ |
| - n | 1,773 | 232 |  |
| - High-intensity | 909 (50.7%) | 112 (48.3%) |  |
| - Moderate-intensity | 883 (49.3%) | 120 (51.7%) |  |
| Diet intervention group |  |  | 0.342^2^ |
| - n | 1,773 | 232 |  |
| - High-protein diet | 883 (49.3%) | 122 (52.6%) |  |
| - Moderate-protein diet | 909 (50.7%) | 110 (47.4%) |  |
| PAL (at BL) |  |  | 0.044^1^ |
| - n | 1,679 | 232 |  |
| - Mean (SD) | 1.62 (0.06) | 1.63 (0.08) |  |
| Sedentary time (at BL) |  |  | 0.121^1^ |
| - n | 1,679 | 232 |  |
| - Mean (SD) | 589.00 (96.46) | 599.28 (79.02) |  |
| LPA (at baseline) |  |  | 0.008^1^ |
| - n | 1,679 | 232 |  |
| - Mean (SD) | 313.05 (83.65) | 297.67 (73.68) |  |
| MVPA (at baseline) |  |  | < 0.001^1^ |
| - n | 1,679 | 232 |  |
| - Mean (SD) | 27.87 (19.62) | 33.09 (24.45) |  |
| PAL (at 6 months) |  |  | < 0.001^1^ |
| - n | 1,081 | 232 |  |
| - Mean (SD) | 1.64 (0.07) | 1.66 (0.08) |  |
| SED (at 6 months) |  |  | 0.694^1^ |
| - n | 1,081 | 232 |  |
| - Mean (SD) | 576.35 (86.34) | 573.91 (83.07) |  |
| LPA (at 6 months) |  |  | 0.631^1^ |
| - n | 1,081 | 232 |  |
| - Mean (SD) | 318.90 (82.29) | 321.72 (76.18) |  |
| MVPA (at 6 months) |  |  | < 0.001^1^ |
| - n | 1,081 | 232 |  |
| - Mean (SD) | 34.47 (23.38) | 40.38 (24.93) |  |
| PAL (at 12 months) |  |  | 0.018^1^ |
| - n | 810 | 232 |  |
| - Mean (SD) | 1.64 (0.07) | 1.65 (0.09) |  |
| SED (at 12 months) |  |  | 0.042^1^ |
| - n | 810 | 232 |  |
| - Mean (SD) | 575.98 (87.13) | 589.00 (81.20) |  |
| LPA (at 12 months) |  |  | 0.044^1^ |
| - n | 810 | 232 |  |
| - Mean (SD) | 314.08 (78.78) | 302.37 (75.40) |  |
| MVPA (at 12 months) |  |  | < 0.001^1^ |
| - n | 810 | 232 |  |
| - Mean (SD) | 33.82 (23.63) | 39.92 (27.05) |  |

^1^ Linear Model ANOVA, ^2^ Pearson’s Chi-squared test, PA - physical activity, BMI – Body Mass Index, BL - Baseline, PAL – physical activity level; SED – sedentary time, LPA – light physical activity; MVPA – moderate-to-vigorous physical activity.
