# Supplementary material for: Combining diaries and accelerometers to explain change in physical activity during a lifestyle intervention for adults with pre-diabetes: A PREVIEW sub-study
Source: PLoS One. 2024 Mar 21;19(3):e0300646. doi: 10.1371/journal.pone.0300646 (PMC10956823; doi:10.1371/journal.pone.0300646)
Supplement: S2 Table — 1 Linear Model ANOVA; 2 Pearson’s Chi-squared test. (DOCX) [file pone.0300646.s004.docx]

**S2 Table. Distribution of age, gender, city, and intervention group between the baseline clusters.**

|  | Cycling cluster  (n = 23) | Walking and housework cluster  (n = 61) | Inactive cluster  (n = 106) | Supervised sports cluster  (n = 42) | Total (n = 232) | p value |
| --- | --- | --- | --- | --- | --- | --- |
| Age, in years (mean (SD)) | 55.6 (12.0) | 57.3 (8.3) | 55.2 (9.4) | 55.9 (10.2) | 55.9 (9.5) | 0.588^1^ |
| Sex (N (%)) |  |  |  |  |  | 0.802^2^ |
| - Female | 13 (56.5%) | 41 (67.2%) | 65 (61.3%) | 26 (61.9%) | 145 (62.5%) |  |
| - Male | 10 (43.5%) | 20 (32.8%) | 41 (38.7%) | 16 (38.1%) | 87 (37.5%) |  |
| Country (N (%)) |  |  |  |  |  | <0.001^2^ |
| - New Zealand | 1 (4.3%) | 3 (4.9%) | 8 (7.5%) | 3 (7.1%) | 15 (6.5%) |  |
| - Denmark | 8 (34.8%) | 4 (6.6%) | 26 (24.5%) | 4 (9.5%) | 42 (18.1%) |  |
| - Finland | 6 (26.1%) | 28 (45.9%) | 44 (41.5%) | 19 (45.2%) | 97 (41.8%) |  |
| - Netherlands | 6 (26.1%) | 1 (1.6%) | 12 (11.3%) | 5 (11.9%) | 24 (10.3%) |  |
| - Spain | 2 (8.7%) | 24 (39.3%) | 11 (10.4%) | 9 (21.4%) | 46 (19.8%) |  |
| - UK | 0 (0.0%) | 1 (1.6%) | 5 (4.7%) | 2 (4.8%) | 8 (3.4%) |  |
| Intervention group (N (%)) |  |  |  |  |  | 0.617^2^ |
| - High intensity group | 13 (56.5%) | 29 (47.5%) | 53 (50.0%) | 17 (40.5%) | 112 (48.3%) |  |
| - Moderate intensity group | 10 (43.5%) | 32 (52.5%) | 53 (50.0%) | 25 (59.5%) | 120 (51.7%) |  |

^1^ Linear Model ANOVA; ² Pearson’s Chi-squared test.
