# Supplementary material for: Combining diaries and accelerometers to explain change in physical activity during a lifestyle intervention for adults with pre-diabetes: A PREVIEW sub-study
Source: PLoS One. 2024 Mar 21;19(3):e0300646. doi: 10.1371/journal.pone.0300646 (PMC10956823; doi:10.1371/journal.pone.0300646)
Supplement: S3 Table — 1 Linear Model ANOVA, PA–physical activity. (DOCX) [file pone.0300646.s005.docx]

**S3 Table. Diary activity z-scores for the baseline clusters.**

|  | Cycling cluster  (n = 23) | Walking and housework cluster  (n = 61) | Inactive cluster  (n = 106) | Supervised sports cluster  (n = 42) | p value |
| --- | --- | --- | --- | --- | --- |
| Walking | -0.36 (0.46) | 0.82 (1.43) | -0.34 (0.49) | -0.15 (0.71) | < 0.001^1^ |
| Cycling | 2.44 (1.58) | -0.35 (0.24) | -0.29 (0.30) | -0.09 (0.48) | < 0.001^1^ |
| Unsupervised sports | 0.50 (2.16) | -0.13 (0.49) | -0.03 (0.86) | -0.01 (0.82) | 0.077^1^ |
| Supervised sports | -0.12 (0.94) | -0.36 (0.54) | -0.42 (0.37) | 1.65 (0.97) | < 0.001^1^ |
| Housework | -0.28 (0.69) | 0.88 (1.32) | -0.47 (0.41) | 0.06 (0.79) | < 0.001^1^ |
| Occupational PA | 0.59 (1.98) | -0.08 (0.62) | -0.09 (0.75) | 0.01 (1.14) | 0.024^1^ |
| Gardening | -0.07 (0.63) | 0.15 (1.57) | -0.05 (0.71) | -0.06 (0.70) | 0.578^1^ |
| Sitting | -0.13 (1.06) | -0.88 (0.62) | 0.57 (0.84) | -0.09 (0.82) | < 0.001^1^ |

^1^ Linear Model ANOVA, PA – physical activity.
