# Supplementary material for: Combining diaries and accelerometers to explain change in physical activity during a lifestyle intervention for adults with pre-diabetes: A PREVIEW sub-study
Source: PLoS One. 2024 Mar 21;19(3):e0300646. doi: 10.1371/journal.pone.0300646 (PMC10956823; doi:10.1371/journal.pone.0300646)
Supplement: S4 Table — (DOCX) [file pone.0300646.s006.docx]

**S4 Table. Distribution of unsupervised and supervised sports between clusters differentiated by type of activity for the baseline clusters in minutes·day^-1^.**

|  | Cycling cluster  (n = 23) | Walking and housework cluster  (n = 61) | Inactive cluster  (n = 106) | Supervised sports cluster  (n = 42) | Total (n = 232) |
| --- | --- | --- | --- | --- | --- |
| **Unsupervised sports** | **11.69 (32.31)** | **2.33 (7.39)** | **3.82 (12.91)** | **4.05 (12.31)** | **4.25 (14.93)** |
| Jogging/running | 1.46 (4.11) | 0.35 (2.05) | 0.32 (1.70) | 0.57 (2.12) | 0.49 (2.22) |
| Water activities | 3.52 (8.53) | 1.68 (6.68) | 1.72 (5.57) | 1.99 (6.02) | 1.94 (6.27) |
| Winter activities | 6.34 (30.38) | 0.30 (2.38) | 1.78 (11.74) | 0.00 (0.00) | 1.52 (12.46) |
| Fishing/hunting | 0.37 (1.79) | 0.00 (0.00) | 0.00 (0.00) | 1.49 (6.83) | 0.31 (2.98) |
| **Supervised sports** | **4.94 (9.27)** | **2.56 (5.30)** | **1.96 (3.65)** | **22.29 (9.53)** | **6.09 (9.83)** |
| (Team) sports | 0.93 (2.49) | 0.46 (3.11) | 0.11 (1.17) | 3.87 (8.43) | 0.97 (4.27) |
| Gymnastics | 4.01 (8.54) | 1.76 (4.16) | 1.70 (3.34) | 14.28 (11.45) | 4.22 (7.92) |
| Dancing | 0.00 (0.00) | 0.34 (1.89) | 0.15 (1.08) | 4.15 (8.26) | 0.91 (3.99) |
