# Supplementary material for: Combining diaries and accelerometers to explain change in physical activity during a lifestyle intervention for adults with pre-diabetes: A PREVIEW sub-study
Source: PLoS One. 2024 Mar 21;19(3):e0300646. doi: 10.1371/journal.pone.0300646 (PMC10956823; doi:10.1371/journal.pone.0300646)
Supplement: S5 Table — SED, LPA & MVPA are reported in mean (SD) minutes per day. Bold indicates significant differences in the post-hoc analyses between groups with the same superscript letters. PAL: physical activity levels, SED: sedentary time, LPA: light physical activity, MVPA: moderate-to-vigorous physical activity. (DOCX) [file pone.0300646.s007.docx]

**S5 Table. Accelerometer-assessed physical activity and sedentary time in the baseline clusters.**

|  | Inactive cluster  (n = 106) | Cycling cluster  (n = 23) | Walking and housework cluster  (n = 61) | Supervised sports cluster  (n = 42) | Total (n = 232) |
| --- | --- | --- | --- | --- | --- |
| PAL | **1.609^a^ (0.054)** | 1.636 (0.057) | **1.663^a^ (0.094)** | 1.637 (0.087) | 1.631 (0.076) |
| SED | **627.3^b^ (69.0)** | 579.2 (97.2) | **565.6^b^ (78.8)** | 588.5 (69.0) | 599.3 (79.0) |
| LPA | **278.0^cd^ (65.2)** | **327.8^c^ (95.3)** | **320.8^d^ (71.2)** | 297.2 (71.4) | 297.7 (73.7) |
| MVPA | **27.2^e^ (17.2)** | 31.18 (19.10) | **43.7^e^ (31.4)** | 33.5 (26.8) | 33.1 (24.5) |

SED, LPA & MVPA are reported in mean (SD) minutes per day. Bold indicates significant differences in the *post-hoc* analyses between groups with the same superscript letters. PAL: physical activity levels, SED: sedentary time, LPA: light physical activity, MVPA: moderate-to-vigorous physical activity.
