# Supplementary material for: Combining diaries and accelerometers to explain change in physical activity during a lifestyle intervention for adults with pre-diabetes: A PREVIEW sub-study
Source: PLoS One. 2024 Mar 21;19(3):e0300646. doi: 10.1371/journal.pone.0300646 (PMC10956823; doi:10.1371/journal.pone.0300646)
Supplement: S6 Table — 1 Linear Model ANOVA; 2 Pearson’s Chi-squared test. (DOCX) [file pone.0300646.s008.docx]

**S5 Table. Distribution of age, gender, city, and intervention group between the baseline to 6 months change clusters.**

|  | Increased walking cluster (n = 73) | Increased supervised sports cluster (n = 87) | Increased cycling cluster (n = 29) | Increased housework cluster (n = 43) | Total (n = 232) | p value |
| --- | --- | --- | --- | --- | --- | --- |
| Age, in years (mean (SD) | 55.5 (9.5) | 55.4 (10.1) | 57.3 (8.3) | 56.6 (9.5) | 55.9 (9.5) | 0.762^1^ |
| Sex (N (%)) |  |  |  |  |  | 0.204^2^ |
| - Female | 46 (63.0%) | 58 (66.7%) | 20 (69.0%) | 21 (48.8%) | 145 (62.5%) |  |
| - Male | 27 (37.0%) | 29 (33.3%) | 9 (31.0%) | 22 (51.2%) | 87 (37.5%) |  |
| Country (N (%)) |  |  |  |  |  | 0.003^2^ |
| - New Zealand | 4 (5.5%) | 10 (11.5%) | 0 (0.0%) | 1 (2.3%) | 15 (6.5%) |  |
| - Denmark | 15 (20.5%) | 11 (12.6%) | 12 (41.4%) | 4 (9.3%) | 42 (18.1%) |  |
| - Finland | 28 (38.4%) | 44 (50.6%) | 10 (34.5%) | 15 (34.9%) | 97 (41.8%) |  |
| - Netherlands | 5 (6.8%) | 8 (9.2%) | 3 (10.3%) | 8 (18.6%) | 24 (10.3%) |  |
| - Spain | 16 (21.9%) | 13 (14.9%) | 4 (13.8%) | 13 (30.2%) | 46 (19.8%) |  |
| - UK | 5 (6.8%) | 1 (1.1%) | 0 (0.0%) | 2 (4.7%) | 8 (3.4%) |  |
| Intervention group (N (%)) |  |  |  |  |  | 0.298^2^ |
| - High intensity group | 32 (43.8%) | 44 (50.6%) | 18 (62.1%) | 18 (41.9%) | 112 (48.3%) |  |
| - Moderate intensity group | 41 (56.2%) | 43 (49.4%) | 11 (37.9%) | 25 (58.1%) | 120 (51.7%) |  |

^1^ Linear Model ANOVA; ² Pearson’s Chi-squared test.
