# Supplementary material for: Combining diaries and accelerometers to explain change in physical activity during a lifestyle intervention for adults with pre-diabetes: A PREVIEW sub-study
Source: PLoS One. 2024 Mar 21;19(3):e0300646. doi: 10.1371/journal.pone.0300646 (PMC10956823; doi:10.1371/journal.pone.0300646)
Supplement: S7 Table — 1 Linear Model ANOVA, PA–physical activity (DOCX) [file pone.0300646.s009.docx]

**S6 Table. Diary activity change z-scores for the baseline to 6 months change clusters.**

|  | Increased walking cluster (n = 73) | Increased supervised sports cluster (n = 87) | Increased cycling cluster (n = 29) | Increased housework cluster (n = 43) | p value |
| --- | --- | --- | --- | --- | --- |
| Walking | 0.51 (0.84) | -0.19 (0.75) | 0.25 (1.03) | -0.65 (1.20) | < 0.001^1^ |
| Cycling | -0.13 (0.36) | -0.33 (0.57) | 1.76 (1.02) | -0.30 (1.20) | < 0.001^1^ |
| Unsupervised sports | 0.17 (0.78) | -0.18 (1.38) | -0.03 (0.30) | 0.08 (0.64) | 0.157^1^ |
| Supervised sports | -0.27 (0.63) | 0.43 (1.34) | -0.04 (0.46) | -0.39 (0.57) | < 0.001^1^ |
| Housework | -0.08 (0.75) | -0.36 (0.58) | -0.35 (1.08) | 1.10 (1.22) | < 0.001^1^ |
| Occupational PA | 0.25 (1.19) | -0.10 (0.50) | 0.18 (0.98) | -0.34 (1.28) | 0.009^1^ |
| Gardening | 0.21 (0.88) | -0.23 (0.67) | 0.17 (1.01) | -0.00 (1.54) | 0.033^1^ |
| Sitting | -0.82 (0.86) | 0.67 (0.69) | -0.09 (0.67) | 0.09 (0.92) | < 0.001^1^ |

^1^ Linear Model ANOVA, PA – physical activity.
