# Supplementary material for: Combining diaries and accelerometers to explain change in physical activity during a lifestyle intervention for adults with pre-diabetes: A PREVIEW sub-study
Source: PLoS One. 2024 Mar 21;19(3):e0300646. doi: 10.1371/journal.pone.0300646 (PMC10956823; doi:10.1371/journal.pone.0300646)
Supplement: S8 Table — (DOCX) [file pone.0300646.s010.docx]

**S7 Table. Distribution of unsupervised and supervised sports change between clusters differentiated by type of activity for the baseline to 6 months change clusters in minutes**·**day^-1^.**

|  | Increased walking cluster (n = 73) | Increased social sports cluster (n = 87) | Increased cycling cluster (n = 29) | Increased housework cluster (n = 43) | Total (n = 232) |
| --- | --- | --- | --- | --- | --- |
| **Unsupervised sports** | **3.75 (13.13)** | **-2.14 (23.17)** | **0.31 (5.00)** | **2.20 (10.72)** | **0.82 (16.85)** |
| Jogging/running | 1.79 (7.01) | 0.95 (4.72) | 1.15 (4.21) | 3.42 (8.82) | 1.70 (6.38) |
| Water activities | -0.39 (5.40) | 0.51 (9.28) | -0.85 (2.77) | -1.11 (7.17) | -0.24 (7.20) |
| Winter activities | 0.94 (8.20) | -3.68 (20.05) | 0.01 (1.74) | -0.12 (0.76) | -1.10 (13.24) |
| Fishing/hunting | 1.41 (8.93) | 0.07 (1.52) | 0.00 (0.00) | 0.00 (0.00) | 0.47 (5.11) |
| **Supervised sports** | **-1.90 (12.03)** | **11.45 (25.62)** | **2.42 (8.77)** | **-4.19 (10.89)** | **3.22 (19.09)** |
| (Team) sports | -0.02 (5.10) | 3.07 (14.23) | 0.67 (3.99) | -1.13 (8.04) | 1.02 (10.00) |
| Gymnastics | -0.78 (9.45) | 6.65 (14.89) | 0.79 (6.84) | -2.49 (9.66) | 1.89 (12.14) |
| Dancing | -1.10 (4.90) | 1.74 (7.70) | 0.96 (4.82) | -0.58 (4.80) | 0.32 (6.18) |
