# Supplementary material for: Combining diaries and accelerometers to explain change in physical activity during a lifestyle intervention for adults with pre-diabetes: A PREVIEW sub-study
Source: PLoS One. 2024 Mar 21;19(3):e0300646. doi: 10.1371/journal.pone.0300646 (PMC10956823; doi:10.1371/journal.pone.0300646)
Supplement: S9 Table — SED, LPA & MVPA are reported in mean (SD) minutes per day. Bold indicates significant differences in the post-hoc analyses between groups with the same superscript letters. PAL: physical activity levels, SED: sedentary time, LPA: light physical activity, MVPA: moderate-to-vigorous physical activity, PA: physical activity. (DOCX) [file pone.0300646.s011.docx]

**S9 Table. Accelerometer-assessed physical activity and sedentary time at baseline and their change after six months for the six-month change clusters.**

|  | Increased walking cluster (n = 73) | | Increased supervised sports cluster (n = 87) | | Increased cycling cluster (n = 29) | | Increased housework cluster (n = 43) | |
| --- | --- | --- | --- | --- | --- | --- | --- | --- |
|  | Baseline | 6-month change | Baseline | 6-month change | Baseline | 6-month change | Baseline | 6-month change |
| PAL | 1.609 (0.053) | **0.062^ab^ (0.060)** | 1.629 (0.055) | **0.021^a^ (0.061)** | 1.621 (0.052) | 0.026 (0.067) | 1.676 (0.126) | **-0.005^b^ (0.076)** |
| SED | 609.3 (79.1) | **-45.1^c^ (78.6)** | 598.2 (69.1) | **-7.7^c^ (78.6)** | 619.2 (87.2) | -37.5 (69.3) | 571.1 (86.7) | -19.4 (80.2) |
| LPA | 290.6 (76.4) | **39.4^d^ (59.6)** | 302.0 (69.4) | **0.8^de^ (59.7)** | 287.2 (65.5) | **51.9^e^ (51.2)** | 308.0 (82.6) | 26.3 (67.9) |
| MVPA | 26.2 (16.0) | **16.8^f^ (18.5)** | 32.8 (20.4) | 6.2 (21.1) | 32.7 (19.2) | 4.3 (26.5) | 45.6 (39.0) | **-4.7^f^ (28.2)** |

SED, LPA & MVPA are reported in mean (SD) minutes per day. Bold indicates significant differences in the *post-hoc* analyses between groups with the same superscript letters. PAL: physical activity levels, SED: sedentary time, LPA: light physical activity, MVPA: moderate-to-vigorous physical activity, PA: physical activity.
