# Supplementary material for: Combining diaries and accelerometers to explain change in physical activity during a lifestyle intervention for adults with pre-diabetes: A PREVIEW sub-study
Source: PLoS One. 2024 Mar 21;19(3):e0300646. doi: 10.1371/journal.pone.0300646 (PMC10956823; doi:10.1371/journal.pone.0300646)
Supplement: S10 Table — 1 Linear Model ANOVA; 2 Pearson’s Chi-squared test. (DOCX) [file pone.0300646.s012.docx]

**S8 Table. Distribution of age, gender, city, and intervention group between the baseline to 12 months change clusters.**

|  | Increased walking & cycling cluster (n = 86) | No change cluster (n = 117) | Increased supervised sports cluster (n = 29) | Total (n = 232) | p value |
| --- | --- | --- | --- | --- | --- |
| Age, in years (mean (SD) | 57.8 (9.2) | 55.0 (9.2) | 54.0 (11.2) | 55.9 (9.5) | 0.063^1^ |
| Sex (N (%)) |  |  |  |  | 0.076^2^ |
| - Female | 53 (61.6%) | 79 (67.5%) | 13 (44.8%) | 145 (62.5%) |  |
| - Male | 33 (38.4%) | 38 (32.5%) | 16 (55.2%) | 87 (37.5%) |  |
| Country (N (%)) |  |  |  |  | 0.634^2^ |
| - New Zealand | 8 (9.3%) | 4 (3.4%) | 3 (10.3%) | 15 (6.5%) |  |
| - Denmark | 16 (18.6%) | 22 (18.8%) | 4 (13.8%) | 42 (18.1%) |  |
| - Finland | 36 (41.9%) | 49 (41.9%) | 12 (41.4%) | 97 (41.8%) |  |
| - Netherlands | 8 (9.3%) | 13 (11.1%) | 3 (10.3%) | 24 (10.3%) |  |
| - Spain | 13 (15.1%) | 27 (23.1%) | 6 (20.7%) | 46 (19.8%) |  |
| - UK | 5 (5.8%) | 2 (1.7%) | 1 (3.4%) | 8 (3.4%) |  |
| Intervention group (N (%)) |  |  |  |  | 0.991^2^ |
| - High intensity group | 42 (48.8%) | 56 (47.9%) | 14 (48.3%) | 112 (48.3%) |  |
| - Moderate intensity group | 44 (51.2%) | 61 (52.1%) | 15 (51.7%) | 120 (51.7%) |  |

^1^ Linear Model ANOVA; ² Pearson’s Chi-squared test.
