# Supplementary material for: Combining diaries and accelerometers to explain change in physical activity during a lifestyle intervention for adults with pre-diabetes: A PREVIEW sub-study
Source: PLoS One. 2024 Mar 21;19(3):e0300646. doi: 10.1371/journal.pone.0300646 (PMC10956823; doi:10.1371/journal.pone.0300646)
Supplement: S11 Table — 1 Linear Model ANOVA, PA–physical activity. (DOCX) [file pone.0300646.s013.docx]

**S9 Table. Diary activity change z-scores for the baseline to 12 months change clusters.**

|  | Increased walking & cycling cluster (n = 86) | No change cluster (n = 117) | Increased supervised sports cluster (n = 29) | p value |
| --- | --- | --- | --- | --- |
| Walking | 0.37 (0.95) | -0.09 (0.80) | -0.74 (1.37) | < 0.001^1^ |
| Cycling | 0.29 (0.85) | -0.17 (0.99) | -0.17 (1.26) | 0.003^1^ |
| Unsupervised sports | 0.07 (0.79) | 0.12 (0.73) | -0.71 (1.88) | < 0.001^1^ |
| Supervised sports | -0.26 (0.40) | -0.25 (0.49) | 1.80 (1.72) | < 0.001^1^ |
| Housework | 0.13 (0.75) | -0.13 (1.07) | 0.16 (1.29) | 0.121^1^ |
| Occupational PA | -0.00 (0.79) | -0.01 (1.19) | 0.04 (0.70) | 0.979^1^ |
| Gardening | 0.12 (0.81) | -0.08 (1.14) | -0.04 (0.91) | 0.340^1^ |
| Sitting | -0.89 (0.78) | 0.59 (0.64) | 0.27 (0.86) | < 0.001^1^ |

^1^ Linear Model ANOVA, PA – physical activity.
