# Supplementary material for: Combining diaries and accelerometers to explain change in physical activity during a lifestyle intervention for adults with pre-diabetes: A PREVIEW sub-study
Source: PLoS One. 2024 Mar 21;19(3):e0300646. doi: 10.1371/journal.pone.0300646 (PMC10956823; doi:10.1371/journal.pone.0300646)
Supplement: S12 Table — (DOCX) [file pone.0300646.s014.docx]

**S10 Table. Distribution of unsupervised and supervised sports change between clusters differentiated by type of activity for the baseline to 12 months change clusters in minutes·day^-1^.**

|  | Increased walking & cycling cluster (n = 86) | No change cluster (n = 117) | Increased social sports cluster (n = 29) | Total (n = 232) |
| --- | --- | --- | --- | --- |
| **Unsupervised sports** | **1.41 (9.06)** | **2.04 (8.42)** | **-7.56 (21.58)** | **0.61 (11.49)** |
| Jogging/running | 1.11 (3.72) | 1.89 (6.44) | -0.40 (4.52) | 1.31 (5.38) |
| Water activities | 0.10 (7.11) | 0.21 (5.03) | -1.91 (8.57) | -0.10 (6.38) |
| Winter activities | 0.37 (2.55) | -0.24 (2.71) | -4.95 (21.67) | -0.60 (8.11) |
| Fishing/hunting | -0.16 (3.06) | 0.17 (1.85) | -0.30 (1.59) | -0.01 (2.35) |
| **Supervised sports** | **0.89 (9.17)** | **0.99 (11.11)** | **47.49 (38.93)** | **6.76 (22.68)** |
| (Team) sports | -0.18 (3.60) | -0.65 (5.43) | 21.81 (48.43) | 2.33 (18.93) |
| Gymnastics | 1.03 (10.28) | 1.22 (8.79) | 18.10 (22.97) | 3.26 (13.16) |
| Dancing | 0.04 (4.56) | 0.42 (5.33) | 7.58 (17.00) | 1.17 (7.93) |
