# Supplementary material for: Combining diaries and accelerometers to explain change in physical activity during a lifestyle intervention for adults with pre-diabetes: A PREVIEW sub-study
Source: PLoS One. 2024 Mar 21;19(3):e0300646. doi: 10.1371/journal.pone.0300646 (PMC10956823; doi:10.1371/journal.pone.0300646)
Supplement: S13 Table — SED, LPA & MVPA are reported in mean (SD) minutes per day. PAL: physical activity levels, SED: sedentary time, LPA: light physical activity, MVPA: moderate-to-vigorous physical activity, PA: physical activity. (DOCX) [file pone.0300646.s015.docx]

**S13 Table. Accelerometer-assessed physical activity and sedentary time at baseline and their change after 12 months for the 12-month change clusters.**

|  | No change cluster (n = 117) | | Increased walking & cycling cluster (n = 86) | | Increased supervised sports cluster (n = 29) | |
| --- | --- | --- | --- | --- | --- | --- |
|  | Baseline | 12-month change | Baseline | 12-month change | Baseline | 12-month change |
| PAL | 1.631 (0.072) | 0.011 (0.071) | 1.670 (0.071) | 0.036 (0.067) | 1.670 (0.071) | 0.016 (0.069) |
| SED | 593.7 (78.6) | -7.1 (84.2) | 582.6 (87.3) | -17.5 (60.6) | 582.6 (87.3) | -1.9 (65.8) |
| LPA | 302.8 (67.8) | -0.2 (54.8) | 303.8 (85.2) | 10.4 (53.1) | 303.8 (85.2) | 7.9 (54.3) |
| MVPA | 32.9 (22.9) | 4.1 (22.6) | 47.6 (24.6) | 10.8 (19.4) | 47.6 (24.6) | 6.2 (25.9) |

SED, LPA & MVPA are reported in mean (SD) minutes per day. PAL: physical activity levels, SED: sedentary time, LPA: light physical activity, MVPA: moderate-to-vigorous physical activity, PA: physical activity.
