# Supplementary material for: Combining diaries and accelerometers to explain change in physical activity during a lifestyle intervention for adults with pre-diabetes: A PREVIEW sub-study
Source: PLoS One. 2024 Mar 21;19(3):e0300646. doi: 10.1371/journal.pone.0300646 (PMC10956823; doi:10.1371/journal.pone.0300646)
Supplement: S14 Table — Bonferroni-adjusted α = 0.0167 to account for multiple testing; significant results are bold. (DOCX) [file pone.0300646.s016.docx]

**S11 Table. Change of device-based PA from baseline to 12 months for the change clusters (Results of individual one-sample t-tests).**

|  | Increased walking & cycling cluster (n = 86) | No change cluster (n = 117) | Increased supervised sports cluster (n = 29) |
| --- | --- | --- | --- |
| PAL | ***t*(85), = 5.02 *p* < 0.001** | *t*(116) = 1.73, p = 0.087 | *t*(28) = 1.22, *p* = 0.233 |
| SED | ***t*(85) = -2.68, *p* = 0.009** | *t*(116) = -0.91, p = 0.367 | *t*(28) = -0.16, *p* = 0.875 |
| LPA | *t*(85) = 1.81, *p* = 0.074 | *t*(116) = -0.05, p = 0.964 | *t*(28) = 0.78, *p* = 0.443 |
| MVPA | ***t*(85) = 5.17, *p* <0.001** | *t*(116) = 1.96, p = 0.052 | *t*(28) = 1.28, *p* = 0.210 |

Bonferroni-adjusted α = 0.0167 to account for multiple testing; significant results are bold.
